# PARP inhibitor‐based treatment in metastatic, castration‐resistant prostate cancer (mCRPC): A systematic review and meta‐analysis

**DOI:** 10.1002/bco2.455

**Published:** 2025-01-14

**Authors:** Michela Roberto, Mattia Alberto Di Civita, Daniele Marinelli, Andrea Torchia, Nertila Cara, Giulia Maltese, Iolanda Speranza, Daniele Santini

**Affiliations:** ^1^ Division of Medical Oncology A Policlinico Umberto I Rome Italy; ^2^ Department of Radiological, Oncological and Anatomopathological Sciences Sapienza University Rome Italy; ^3^ Department of Experimental Medicine Sapienza University Rome Italy; ^4^ Division of Medical Oncology B Policlinico Umberto I Rome Italy; ^5^ Department of Medical‐Surgical Sciences and Biotechnologies Sapienza University Latina Italy

**Keywords:** androgen receptor signalling inhibitor (ARSI), androgen receptor‐targeted agents (ARTA), BRCA, DNA damage repair defects (DDR), homologous recombination repair (HRR), poly‐ADP ribose polymerase (PARP) inhibitors, prostate cancer (PCa)

## Abstract

**Background:**

We present a systematic review and meta‐analysis of randomized clinical trials (RCTs) with PARPi either as monotherapy or in combination with an androgen receptor‐targeted agent (ARTA) in first‐ and second‐line settings.

**Methods:**

Primary endpoints are radiographic progression free survival (rPFS) and overall survival (OS) in patients with mCRPC and either unselected, homologous recombination repair wild‐type (HRR−), homologous recombination repair mutated (HRR+) or with BRCA1, BRCA2, or ATM mutation. The effect of PARPi + ARTA in the second‐line setting is also explored. Safety is a secondary end‐point.

**Results:**

A total of five phase III (first line: MAGNITUDE, PROpel, TALAPRO‐2; second line: PROfound, TRITON3) and two phase II RCTs (second line: NCT01972217, NCT01576172) were selected. In the first‐line setting, rPFS was significantly improved in PARPi + ARTA arm in all comers (HR 0.70, *p* < 0.00001), HRR− (HR 0.76, *p* = 0.005), HRR+ (HR 0.57, *p* = 0.0003), and BRCA1/2‐mutated patients (HR: 0.33, *p* < 0.00001). OS was improved in the population with HRR+ status (HR 0.76, *p* = 0.02) but not statistically significant in BRCA1/2‐mutated patients (HR 0.57, 95% CI 0.30–1.08, *p* = 0.08). In the second line, PARPi improves rPFS (HR for BRCA2 0.31, *p* = 0.002) and OS (HR for BRCA1/2 0.71, *p* = 0.01) only in such patients. In this setting, no advantage was reported by adding a PARPi to an ARTA. The arm with PARPi either as monotherapy or in combination with ARTA showed a significantly higher toxicity profile.

**Conclusions:**

PARPi‐based therapy represents a compelling treatment option for HRR+ mCRPC, mainly BRCA1/2‐mutated patients. However, further biomarker analysis are needed in order to identify other responsive patients across the different disease settings.

## 1 INTRODUCTION

Prostate cancer (PCa) is the second most common neoplasm in males worldwide, affecting 14.3% of the male population.^1^ Despite recent therapeutic advances, metastatic prostate cancer (mPC) is an incurable disease with a 5‐year survival rate of 31%.^2^

The mainstay treatment of mPC is androgen‐deprivation therapy (ADT); however, patients treated with ADT alone will inevitably go on to develop castration‐resistant prostate cancer (mCRPC). Over the last two decades, multiple hormone therapies, chemotherapies, and radioligands were shown to improve overall survival (OS) in mCRPC.^3^, ^4^, ^5^, ^6^, ^7^, ^8^

The BRCA1 and BRCA2 proteins are essential to repair DNA double‐strand breaks (DSBs) through the homologous recombination repair (HRR) mechanism.

Germline mutations in the BRCA1 and BRCA2 genes were present in 0.5%–2% of patients with PCa and were associated with more aggressive features and worse prognosis in PCa, while somatic mutations in BRCA1 or BRCA2 were present in 15%–25% of mCRPC.^9^, ^10^, ^11^, ^12^ Additionally, mutations in HRR genes were found in almost 30% of screened cases mCRPC cases in the PROfound study.^13^

Poly(adenosine diphosphate [ADP]–ribose) polymerase (PARP) enzymes are crucial in the DNA damage response (DDR) by recruiting DNA repair effectors. Their inhibition through the PARP inhibitors (PARPi) leads to the accumulation of DNA damage that, in cells with DDR pathway deficiencies such as BRCA1/2 mutations, causes cell death, a mechanism known as synthetic lethality.^14^

The PARPi olaparib, rucaparib, niraparib, and talazoparib showed promising activity in mCRPC with somatic or germline alterations in BRCA1, BRCA2 or other HRR genes in phase II‐III randomized clinical trials (RCTs).^15^, ^16^, ^17^, ^18^, ^19^, ^20^, ^21^, ^22^, ^23^, ^24^, ^25^, ^26^, ^27^, ^28^, ^29^ More recently, PARPi were tested in unselected mCRPC in combination with androgen receptor‐targeted agents (ARTA). We performed a systematic review and meta‐analysis of randomized clinical trials (RCTs) assessing the efficacy of PARPi in mCRPC either alone or in combination with ARTA.

## 2 METHODS

### 2.1 Included studies

**FIGURE 1:**
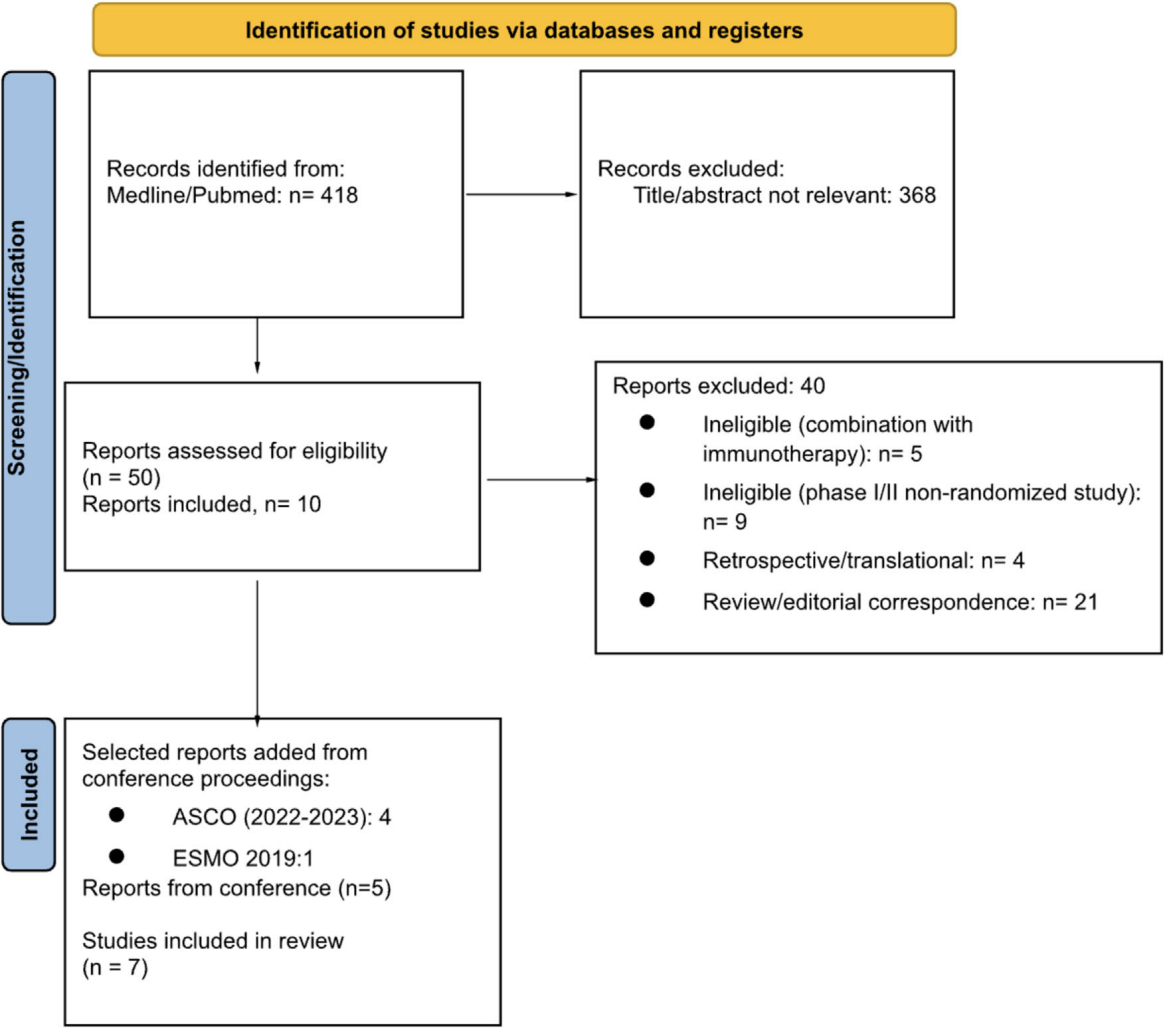
PRISMA flow diagram of the study.

We included phase III and II‐RCTs reporting on efficacy and safety of PARP inhibitor‐based treatment, alone or in combination with an ARTAin mCRPC (Figure 1). The control arm was either an ARTA alone or treatment of physician's choice (TPC), including chemotherapy in second‐line settings. Nonrandomized, early phase clinical trials were excluded from the present analysis. Only studies published in the English language were included.

### 2.2 Search strategy and selection processes

A systematic literature search was conducted on the MEDLINE, EMBASE, and CENTRAL databases in June 2023 independently by two authors (MADC and DM). We used Medical Subject Headings (MeSH) for the MEDLINE and CENTRAL databases, Emtree for the EMBASE database, and selected keywords; the results were filtered by including only reports on clinical trials or randomized clinical trials.

For each outcome, the most recent available report from each clinical trial was included. Whenever judged appropriate by the first authors, we included results from unpublished online data matched to meeting abstracts.

The following variables were extracted from all included studies: first authors, year of first publication, study name (NCT number), eligibility criteria, treatment arms, number of patients enrolled in each arm, number of patients with a mutation in homologous‐recombination repair (HRR) genes, number of patients with a BRCA1/2 gene mutation, previous exposure to ARTA or to chemotherapy for mPC, and study endpoints.

Radiological PFS (rPFS) was defined as the time from randomisation to imaging‐based progression or death, whichever occurred first; blinded independent central review (BICR) or investigator‐assessed analyses were included based on the included studies' primary endpoint. OS was defined as the time from randomisation to death.

Safety was assessed with any grade and grade ≥3 treatment‐related adverse events. Adverse events (AEs) grading was performed with the Common Terminology Criteria for Adverse Events (CTCAE) criteria version 4 or 5, depending on the included study.

### 2.3 Endpoints

Primary endpoints:

- rPFS and OS in unselected, HRR+, HRR−, and BRCA1/2‐mutated patients in studies assessing the efficacy of a PARP inhibitor combined with an ARTA in the first‐line setting
- rPFS and OS in BRCA1‐, BRCA2‐ or ATM‐mutated patients in studies assessing the efficacy of PARP inhibitor monotherapy in the second‐line setting

Secondary endpoints:

- Safety in both first‐line and second‐line settings studies

Exploratory endpoints:

- rPFS in unselected patients in studies evaluating PARPi with an ARTA in the second‐line setting
- rPFS in patients with bone or visceral metastasis, evaluating the efficacy of a PARP inhibitor combined with an ARTA in the first‐line setting

### 2.4 Statistical analysis

Hazard ratios (HR) with 95% confidence intervals (95% CI) were extracted for survival endpoints; the effect was estimated with a random effects model based on inverse variance. For categorical endpoints, the number of events for each group was collected. Odds ratios (OR) and 95% CI were estimated with a Mantel–Haenszel random effects model. Statistical heterogeneity between studies was quantified with Higgins' *I* ^2^. Comparisons between subgroups were carried out using a *χ* ^2^ test.

This analysis was conducted in accordance with the PRISMA (Preferred Reporting Items for Systematic Reviews and Meta‐Analyses) guidelines (Figure S9). All analyses were performed with RevMan Web version 5.3.1 by two authors (MADC and DM). The *alpha* for all analyses was set at 0.05. The analysis on rPFS in patients with bone or visceral metastasis, evaluating the efficacy of a PARP inhibitor combined with an ARTA in the first‐line setting was performed with R‐Studio software.

### 2.5 Risk of bias assessment

The risk of bias was assessed by two authors (MR and DM) using the RoB2 tool, and disagreements were resolved by a third author (MADC). (Figure S1).

## 3 RESULTS

### 3.1 Included studies

The literature search on the MEDLINE, EMBASE and CENTRAL databases identified 220 records; after the exclusion of duplicates and of reports not matching inclusion criteria, we identified 11 reports from seven studies (PROfound,^19^, ^20^ TRITON3,^21^ PROpel,^22^, ^23^, ^24^ MAGNITUDE,^25^, ^26^ TALAPRO‐2,^27^, ^28^ NCT01972217,^29^ NCT01576172^30^); the flow diagram of the study is shown in Figure 1.

Two of the included studies (PROfound, TRITON3) compared a PARP inhibitor to TPC; five of the included studies compared a PARP inhibitor combined with an ARTA versus ARTA alone in first‐line setting (PROpel, MAGNITUDE, TALAPRO‐2) or in the second‐line setting (NCT01972217, NCT01576172). The PARPi olaparib was tested in three of the included studies (PROfound, PROpel, NCT01972217), while niraparib (MAGNITUDE), talazoparib (TALAPRO‐2), rucaparib (TRITON3), and veliparib (NCT01576172) were tested in one study each.

Characteristics of included studies and enrolled patients are summarized in Table 1.

**TABLE 1:** Summary of clinico‐pathological characteristics of the included studies.

Study (year)	Study name (NCT number)	Eligible patients	Study drug	No. of patients	Previous treatment exposure	No. of HRR+ patients	No. of BRCAm patients	Endpoints
Agarwal et al. 2022	TALAPRO 2 (NCT03395197)	I line mCRPC	Talazoparib + enzalutamide vs. Placebo + enzalutamide	402 vs. 403	Abiraterone (mHSPC): 21 vs. 25 Docetaxel (mHSPC): 86 vs. 93 Both: 109 vs. 110	85 (21.1%) vs. 84 (20.8%)	28 (7%) vs. 32 (8%)	BICR‐rPFS: NR vs. 21.9 months HR 0.63 [0.51–0.78] OS: HR 0.89 ORR: 61.7% vs. 43.9% PFS2: HR 0.77 [0.61–0.98] TT PSA Progression: HR 0.72 [0.58–0.89] TTCC: HR 0.49 [0.38–0.65]
Kim N Chiet al. 2022	MAGNITUDE (NCT03748641)	I line mCRPC HRR+; HRR−	Niraparib + abiraterone vs. Placebo + abiraterone	212 vs. 211	Abiraterone (nmCRPC or mHSPC): 8 vs. 5 Docetaxel (mHSPC): 41 vs. 44	212 vs. 211	113 (53.3%) vs. 112 (53.1%)	BICR‐rPFS: 16.6 months vs. 13.7 months HR 0.73 [0.56–0.96] BICR‐rPFS in BRCAm: 19.5 months vs. 10.9 months HR 0.55 [0.39–0.78] OS in BRCAm: 29.3 months vs. 28.6 months HR 0.96 [0.57–1.67] ORR: 60% vs. 28% TT PSA Progression: HR 0.57 [0.43–0.76] TTCC in BRCAm: HR 0.56 [0.35–0.90]
Fred Saad et al. 2022	PROpel (NCT03732820)	I line mCRPC	Olaparib + abiraterone vs. Placebo + abiraterone	399 vs. 397	Abiraterone: 1 vs. 0 Docetaxel (adj or mHSPC): 97 vs. 98	111 vs. 115	47 vs. 38^a^	IA‐rPFS: 24.8 months vs. 16.6 months HR 0.66 [0.54–0.81] mOS°: 42.1 months vs. 34.7 months HR 0.81[0.67–1.00] ORR: 54% vs. 48.1% PFS2: HR 0.76 [0.59–0.99] TSFT: HR 0.76 [0.64–0.90]
J De Bono et al. 2020	PROFOUND (NCT02987543)	II line mCRPC Cohort A: BRCA1, BRCA2 or ATM; Cohort B: any of 12 other HRR alterations^b^	*Cohort A*: Olaparib vs. ARTA *Cohort B*: Olaparib vs. ARTA	A: 162 vs. 83 B:94 vs. 48	Enza (mCRPC, nmCRPC, mHSPC): 68 vs. 40 Abi (mCRPC, nmCRPC, mHSPC): 62 vs. 29 Enza + Abi: 32 vs. 14 Docet (mHSPC): 74 vs. 32 Cabazit: 2 vs. 0 Doce + Cabaz: 29 vs. 20 Paclitaxel: 1 vs. 0	A: 245 B: 142	A: 101 vs. 57 B: 1 vs. 1	BICR‐rPFS cohort A: 7.4 months vs. 3.6 months HR 0.34 [0.25–0.47]; in ATMm HR 1.04 [0.61–1.87] mOS: 18.5 months vs. 15.1 months HR 0.64 ORR: 33% vs. 2%
K. Fizazi et al. 2023	TRITON 3 (NCT02975934)	II line mCRPC, BRCA1/2 o ATM mut	Rucaparib vs. TPC (docetaxel, enzalutamide, or abiraterone)	270 vs. 135	Enzalutamide (CRPC): 119 vs. 61 Abiraterone (CRPC): 150 vs. 80 Docetaxel (mHSPC): 63 vs. 28 Apalutamide (CRPC): 8 vs. 1	270 vs. 135	201 vs. 101	BICR‐rPFS in BRCAm: 11.2 months vs. 6.4 months HR 0.50 [0.36–0.69]; in ATMm: 8.1 months vs. 6.8 months HR 0.95 [0.59–1.52] in ITT: 10.2 months vs. 6.4 months HR 0.61 [0.47–0.80] OS in BRCAm: 24.3 months vs. 20.8 months HR 0.81 [0.58–1.12] ORR in BRCAm: 45% vs. 17% ORR in ATMm: 0% vs. 14% TTPP: HR 0.44
Clarke et al. 2018	NCT01972217	II line, mCRPC unselected	Abiraterone + olaparib vs. Abiraterone + placebo	71 vs. 71	Docetaxel: 71 vs. 71 Cabazitaxel:10 vs. 9 Abiraterone: 0 vs. 1	11 vs. 10	2 vs. 4	IA‐rPFS 13.8 mo vs. 8.2 months, HR 0.65 (0.44–0.97) mOS 23.3 months vs. 20.9 months HR 0.89 (0.58–1.35) mPFS2: 23.3 months vs. 18.5 months mDOR: 17.8 months vs. 12.1 months
Hussain et al. 2018	NCT01576172	II line mCRPC, unselected	Abiraterone + placebo vs. Abiraterone + veliparib	74 vs. 79	Docet/Cabazit: 11 vs. 17 Other cht: 5 vs. 6 Enzalutamide: 2 vs. 2 Sipuleucel‐T: 22 vs. 13 Experimental agent: 19 vs. 15	13 vs. 7	14 vs. 3	PSA50: 63.9% vs. 72.4% mPFS 10.1 months vs. 11 months mRR 45.0% vs. 52.2%

Abbreviations: ARTA, androgen‐receptor targeted agent; BICR, blinded independent central review; HRR, homologous recombination repair; IA, investigator‐assessed; NHA, new hormonal agent (e.g., enzalutamide or abiraterone); PSA50, 50% or greater decline in PSA from baseline; TFST, time to first subsequent therapy or death; TPC, treatment of physician's choice; TTCC, time to cytotoxic chemotherapy; TTPP, time to pain progression.

^a^ BRIP1, BARD1, CDK12, CHEK1,CHEK2, FANCL, PALB2, PPP2R2A, RAD51B, RAD51C, RAD51D, or RAD54L. Among patients in the control group, 81% crossed over to receive olaparib treatment.

^b^ Overall survival in BRCAm patients HR 0.29.

### 3.2 Efficacy of PARPinhibitors in unselected, HRR+, and HRR− mCRPC and in mCRPC with a BRCA1, BRCA2, or ATM mutation

#### 3.2.1 First‐line setting

**FIGURE 2:**
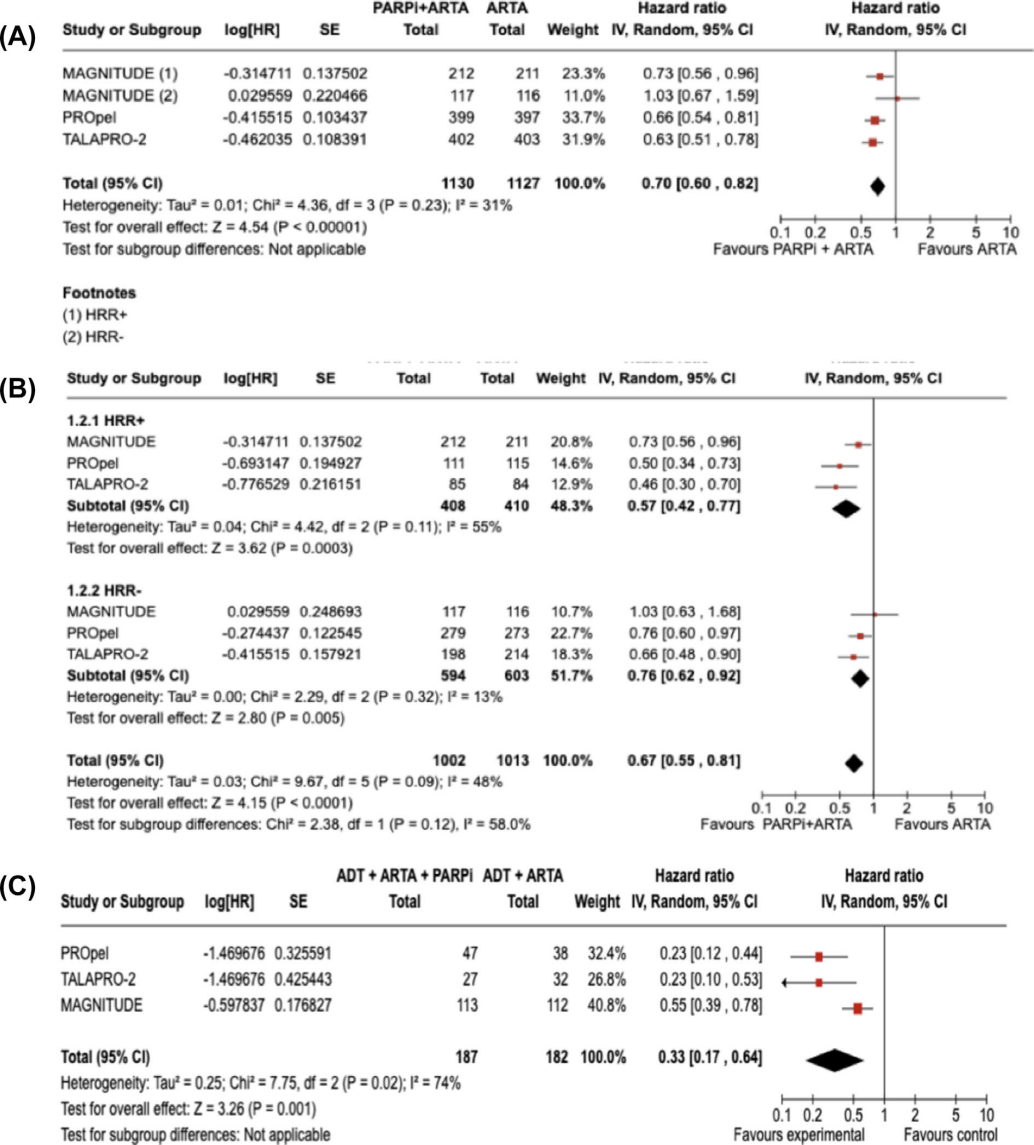
Radiographic progression‐free survival in studies comparing PARPi combined with ARTA compared with ARTA alone; (A) patients with any HRR status (positive, negative or unknown); (B) patients with known HRR status; (C) patients with a BRCA1/2 gene mutation.

Considering 2257 patients with any HRR status (positive, negative or unknown) from the three included studies (MAGNITUDE, PROpel, TALAPRO‐2), the combination of PARPi and ARTA led to improved rPFS when compared with an ARTA alone (HR 0.70, 95% CI 0.60–0.82, *p* < 0.00001; Figure 2A). The presence of alterations in any HRR gene (HRR+) was determined with study‐specific criteria based on different gene lists, gene panels and sequencing methods. The HRR+ and HRR negative (HRR−) categories are therefore based on the classification from each study. Even if the HRR− cohort in the MAGNITUDE study was closed early due to a futility analysis, the results were included in the present analysis. Among 2015 patients with known HRR status, rPFS was improved in the experimental arm both in HRR+ (HR 0.57, 95% CI 0.42–0.77, *p* = 0.0003) and HRR− patients (HR 0.76, 95% CI 0.62–0.92, *p* = 0.005; Figure 2B). Among 465 patients with BRCA1 or BRCA2 gene mutations, rPFS was improved in the experimental arm (HR 0.33, 95% CI 0.17–0.64, *p* < 0.00001; Figure 2C).

**FIGURE 3:**
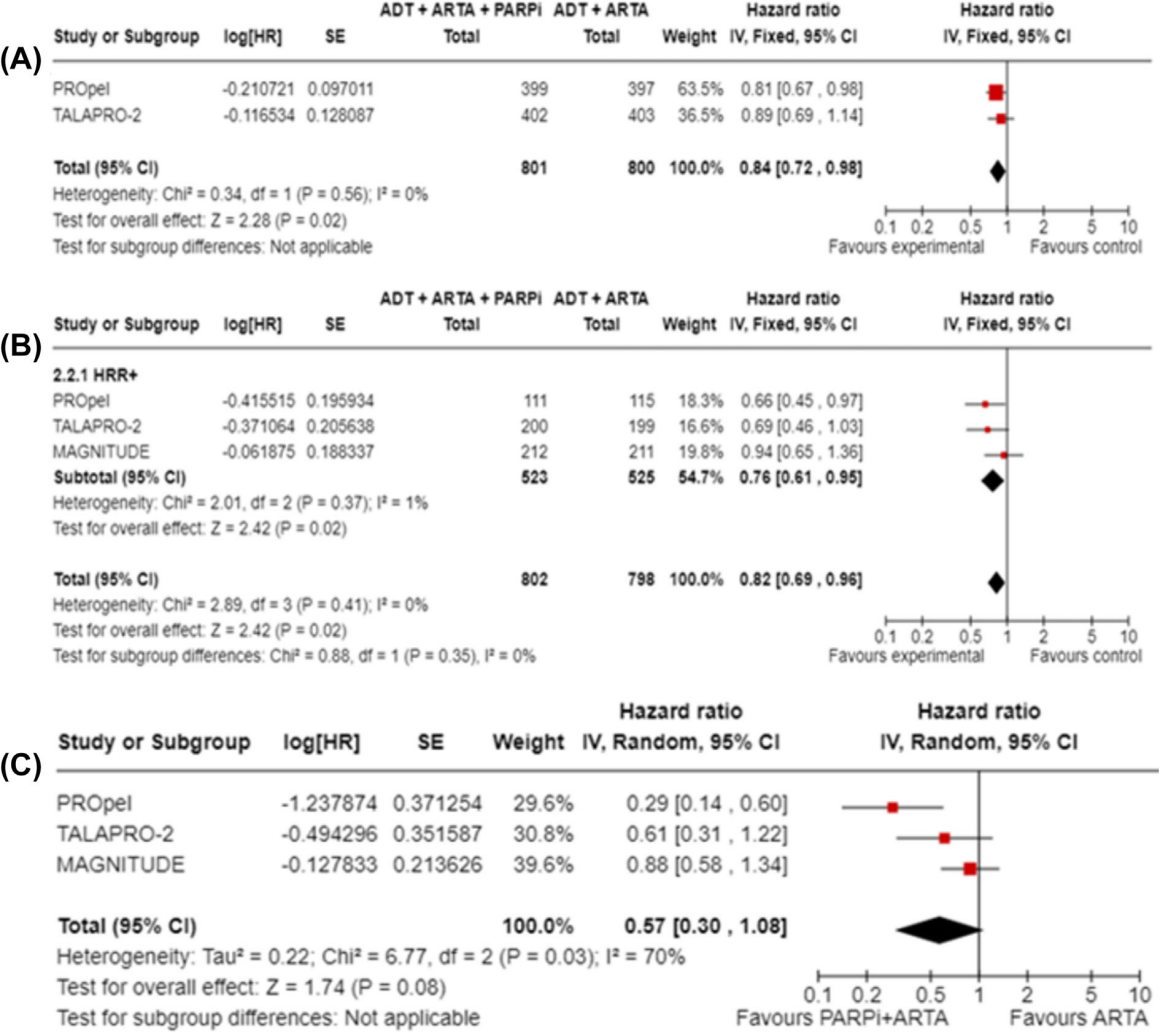
OS in studies comparing PARPi combined with ARTA compared with ARTA alone; (A) unselected patients; (B) patients with HRR+ status; (C) patients with a BRCA1/2 gene mutation.

With the combination of PARPi and ARTA, OS was significantly improved both in HRR+ patients (HR 0.76, 95% CI 0.61–0.95, *p* = 0.02; Figure 3B) and unselected patients (MAGNITUDE not included; HR 0.84, 95% CI 0.72–0.98, *p* = 0.02; Figure 3A). Numerically improved OS was reported in the pooled analysis of BRCA1‐ and BRCA2‐mutated patients (HR 0.57, 95% CI 0.30–1.08, *p* = 0.08; Figure 3C).

The combination of PARPi and ARTA in patients with visceral metastasis improved significantly rPFS versus ARTA only (HR 0.72, 95% CI 0.57–0.91, *p* = 0.0053). In the Patients with bone only metastases, the combination of PARPi + ARTA improved the rPFS compared with ARTA alone (HR: 0.68, 95% CI 0.55–0.84, *p* = 0.0003; Figure S2).

#### 3.2.2 Second‐line setting

**FIGURE 4:**
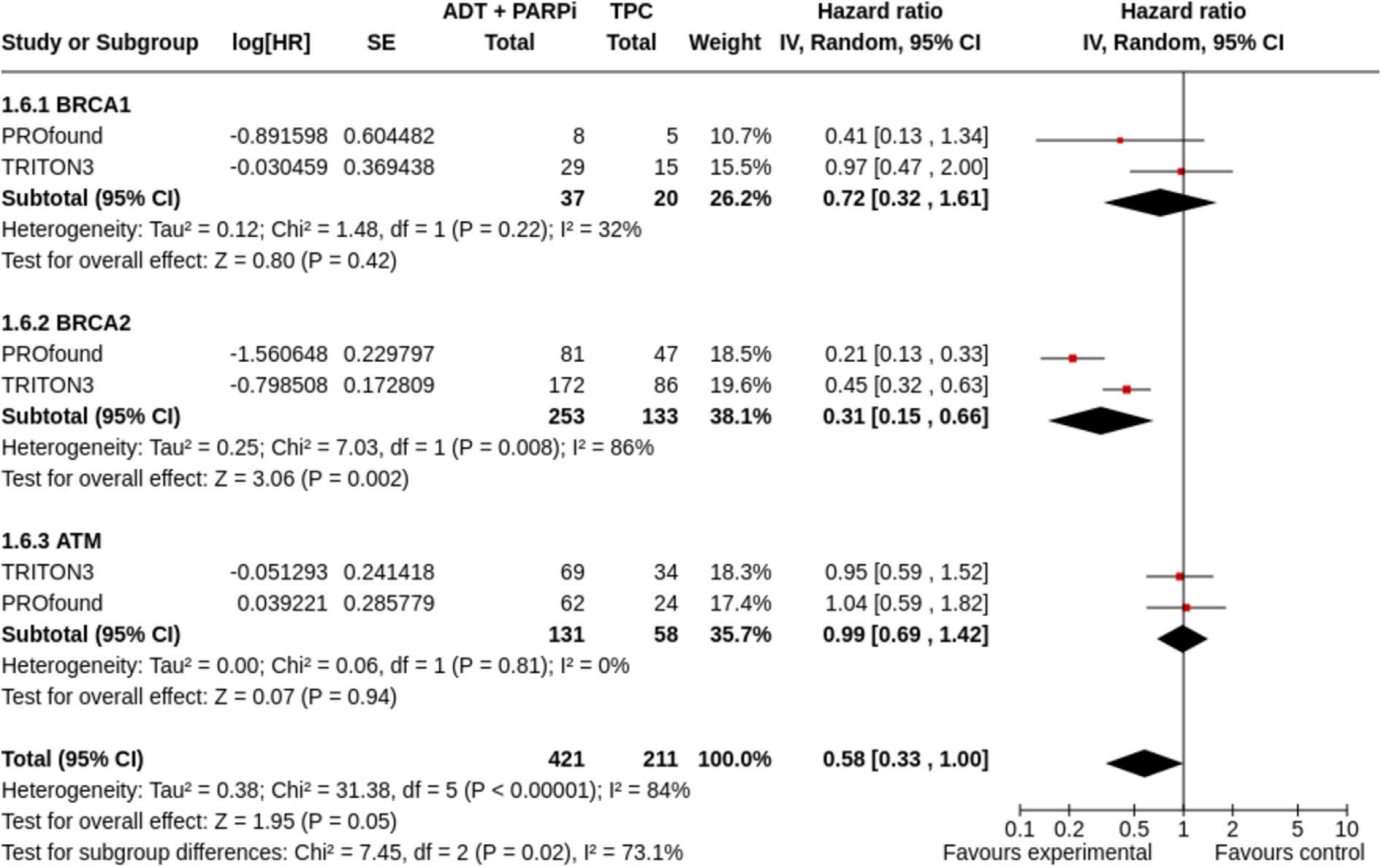
Radiological progression‐free survival in mCRPC with a BRCA1, BRCA2 or ATM mutation in studies comparing PARPi monotherapy to TPC.

**FIGURE 5:**
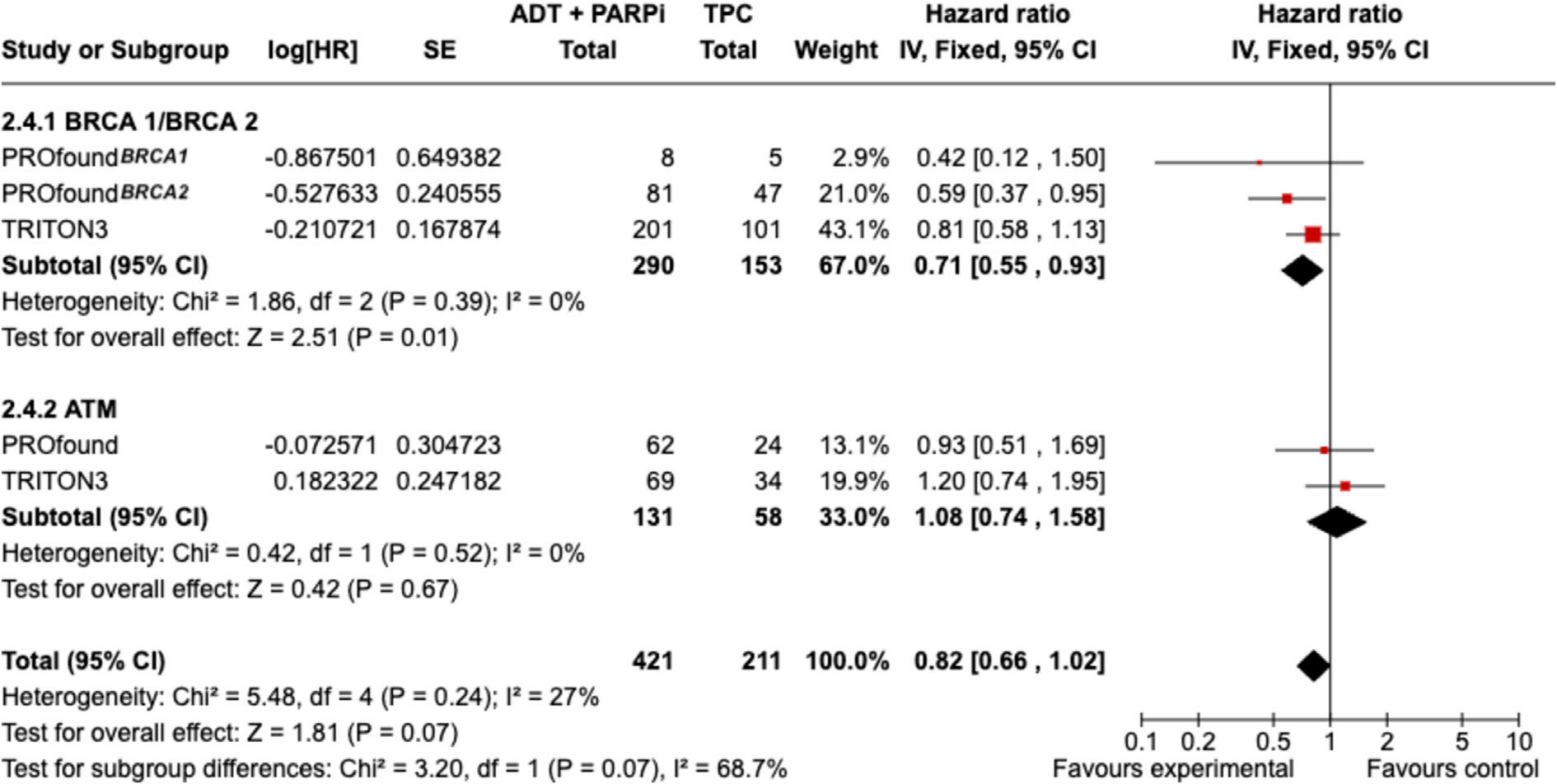
OS in mCRPC with a BRCA1, BRCA2, or ATM mutation in studies comparing PARPi to TPC.

Among 632 patients from two studies (PROfound, TRITON3) with a mutation in BRCA1 (*n* = 57), BRCA2 (*n* = 386), or ATM (*n* = 189), the treatment with PARPi compared with TPC led to improved rPFS only in BRCA2‐mutated patients (HR 0.31, 95% CI 0.15–0.66, *p* = 0.002; Figure 4); pooled OS resulted to be higher in BRCA1/BRCA2 patients (HR = 0.71, 95% CI 0.55–0.93, *p* = 0.01), but it was worse in ATM‐mutated patients (HR 1.08, 95% CI 0.74–1.58, *p* = 0.67; Figure 5).

In terms of rPFS, considering unselected patients, no advantage was reported by adding a PARPi to an ARTA in the included phase II studies. (HR = 0.81, 95% CI 0.53–1.24, *p* = 0.34; Figure S3).

### 3.3 Safety

Considering PARPi in combination with ARTA (MAGNITUDE, PROpel, TALAPRO‐2, NCT01972217, NCT01576172), among 2557 patients, any grade treatment‐related AEs occurred in 1251/1281 (98%) and 1193/1276 (93%) patients in the experimental arm compared with the control arm (OR 2.97, 95% CI 1.92–4.58, *p* < 0.00001; Figure S4). Grade ≥3 AEs occurred in 810 (63%) and in 539 (42%) in the experimental arm compared with ARTA alone (OR 2.36, 95% CI 1.61–3.45, *p* < 0.0001; Figure S5). When PARPi is used as monotherapy (PROfound, TRITON‐3), among 786 patients, any grade AEs occurred in 514/526 (98%) and in 243/260 (93%) patients in the experimental arm compared with the control arm (OR 2.98, 95% CI 1.40–6.37; Figure S6); grade ≥3 AEs occurred in 291 (55%) and in 118 (45%) patients in the experimental arm compared with the control arm (OR 1.49, 95% CI 1.10–2.01, *p* = 0.10; Figure S7).

## 4 DISCUSSION

Despite the latest advances in mCRPC treatment in the past decade thanks to the introduction of taxanes and new hormonal agent (NHA), there is still a high unmet need in this population.^1^, ^2^, ^3^, ^4^, ^5^, ^6^, ^7^, ^8^ Recently, PARP inhibitors, either as monotherapy or in combination with other agents, have been explored in several RCTs with promising results in mCRPC, mainly in patients with alterations in specific genes. Indeed, up to 25% of patients with mPC present a germline or somatic alteration in HRR genes, most frequently BRCA2, which are associated with aggressive disease and poor outcomes but are potentially responsive to targeted agents.^27^, ^31^ Accordingly, the US Food and Drug Administration and the European Medicines Agency, approved rucaparib and olaparib in BRCA‐mutated and HRR‐gene mutated mCRPC, respectively.^2^ However, their use in the current clinical practice is still limited due to the lack of both predictive biomarkers and long‐term survival data. The rationale behind the association of a PARPi and a NHA derives from preclinical studies showing that the inhibition of the androgen pathway leads to the downregulation of the HRR pathway and to the upregulation of the PARP activity. This causes an HRR deficiency‐like state making the cell more dependent on the PARP signalling. Therefore, the concomitant inhibition of the androgen pathway can enhance the activity of PARPi.^32^

The present meta‐analysis differs from other published reports^33^, ^34^, ^35^ because we have thoroughly assessed phase II and III RCTs which have explored the efficacy and safety of PARP inhibitors‐based treatment in mCRPC, both as first‐line (PROpel, MAGNITUDE and TALAPRO‐2) or as second‐line therapy (PROfound and TRITON‐3, NCT01972217, NCT01576172).

In terms of efficacy, our results show that PARPi in combination with an ARTA as first‐line is a compelling treatment option for mCRPC patients, with a significantly improved survival rPFS in the overall population (HR 0.70, 95% CI 0.60–0.82, *p* < 0.000001). However, a higher magnitude of benefit is shown in BRCA‐mutated patients (HR 0.33, 95% CI 0.17–0.64) rather than HRR+ (HR 0.57, 95% CI 0.42–0.77) or HRR− (HR 0.76, 95% CI 0.62–0.92). While OS is significantly improved both overall and in HRR+ patients with an HR of 0.84 and 0.76, respectively, a larger numerical improvement is shown in patients with a BRCA1/2 mutation (i.e., HR 0.57, 95% CI 0.30–1.08); therefore, it is likely that the BRCA1/2‐mutated subgroup is driving the results in HRR+ patients. On the other hand, in the second‐line setting, the addition of a PARPi to an ARTA is not associated with a statistically significant or clinically relevant improvement in rPFS (unselected patients HR 0.81, 95% CI 0.53–1.24). However, when compared with an ARTA/chemotherapy, PARPi monotherapy significantly improved rPFS in the second‐line treatment of mCRPC with a BRCA2 mutation (HR 0.31, 95% CI 0.15–0.66). In terms of OS, even if data are still immature, the PARPi result to be a better option than ARTA alone or chemotherapy in BRCA1/2 patients (HR 0.71, 95% CI 0.55–0.93) but not in patients with ATM mutation (HR 1.08, 95% CI 0.74–1.58). Finally, in terms of safety (secondary end‐point), PARPi, whether in combination with ARTA or as monotherapy, shows an increased toxicity profile compared with the control arms, although mainly hematologically based, as expected, and thus considered manageable across the studies.

However, some considerations should be made.

First of all, the patient population is very different among the three trials in the first‐line setting: the PROpel trial includes all‐comers with HRR status assessed retrospectively^18^; the MAGNITUDE trial includes two distinct cohorts of patients, with or without HRR alterations (and the HRR− cohort was closed early for futility thus we cannot extrapolate results for OS in any HRR or HRR− cohorts), otherwise enrolling specific assessment of patients with BRCA mutations^22^; the TALAPRO‐2 study includes patients unselected for HRR status and the patients' stratification have been done for prior abiraterone or docetaxel in the mHSPC setting.^23^

Additionally, another relevant consideration is related to the fact that, even if data are still immature, these combinations seem to improve OS in the all comers population (PROpel, TALAPRO‐2; HR 0.84, 95% CI 0.72–0.98), or with at least one mutation in HRR genes (MAGNITUDE, PROpel, TALAPRO‐2; HR 0.76, 95% CI 0.61–0.95). While the improvement of OS in patients with a BRCA1/2 mutation was not statistically significant, this selected population has the numerically largest improvement (HR 0.57, 95% CI 0.30–1.08); accordingly, this subgroup analysis was conducted in a limited number of patients; overall, there are 310 BRCA‐mutated patients (MAGNITUDE: 225, PROpel: 85, TALAPRO‐2: 60). Certainly, molecular analysis helps to select patients who benefit most by the addition of a PARPi to hormone therapy, and this is true both in the subgroup of patients with mutations in HRR genes, and even more in tumours with BRCA mutations (where olaparib + abiraterone leads to a 71% reduction in the risk of death). However, the wide confidence interval and the heterogeneity of population in the included studies does not allow us to draw definitive conclusions for OS in this setting.

For the second‐line outcomes, our meta‐analysis considers the phase III trials PROfound and TRITON‐3, which are characterized by the following criteria: (i) Both trials include patients with mCRPC who experienced PD during the treatment with an ARTA and randomly assigned to a PARPi (olaparib and rucaparib, respectively) versus TPC (abiraterone, enzalutamide or docetaxel); (ii) previous chemotherapy with taxane was allowed in the PROfound but not in the TRITON‐3; (iii) in the PROfound, all the patients had an alteration in at least 1 of 15 prespecified HRR genes (cohort A in BRCA1/2 or in ATM, cohort 2 in any of the other 12 genes), whereas the TRITON3 trial includes only patients with a BRCA1/2 or ATM alteration; (iv) in both trials, the experimental arm shows an advantage in terms of rPFS (primary end‐point) in the population with alterations in BRCA1/2 or ATM (PROfound: HR 0.34, 95% CI 0.25, 0.47; TRITON3: HR 0.61, 95% CI 0.47, 0.80). However, evaluating separately BRCA1/2‐ and ATM‐mutated population, this result has been confirmed only in the former and not in the latter. We have also done an exploratory analysis on PARPi combined with abiraterone in second‐line, ARTA‐naive, patients in phase II‐randomized studies, characterized by conflicting results: olaparib + abiraterone (NCT01972217) provided rPFS benefit (HR 0·65, 95% CI 0·44–0·97); veliparib + abiraterone (NCT01576172) did not show survival advantage (HR1.00, 95% CI 0.70–1‐43). But compared with other PARP inhibitors, veliparib demonstrates overall lower levels of PARP trapping activity that may clarify these differences and the lack of approval by FDA (ref. doi.org/10.1016/B978‐0‐323‐77684‐4.00025‐8).

Notably, OS data from the TRITON3 are still immature and are probably influenced by the high evidence of crossing over from the control arm to the experimental one. Furthermore, as recently commented by two experts, the lack of OS improvement in this setting could be due to some methodological pitfalls in both PROfound and TRITON3 trials.^36^

In the present meta‐analysis, we analyse as secondary‐endpoints the safety of PARPi‐based treatments. The pooled data of toxicities registered in the trials with PARPi both as monotherapy (PROfound and TRITON3) or as combining treatment (PROPEL, TALAPRO‐2, MAGNITUDE, NCT01972217, NCT01576172) show a higher incidence of AEs in the PARPi groups compared with the control group. As reported in the Supporting Information, the profile of toxicities is similar to that reported by previous studies,^37^ and the most common AEs are anaemia and thrombocytopenia overall, even if grade >G3 AEs are higher in combination strategies (Figure S8). Pulmonary embolism events have been observed in PARPi combined with ARTA as well as with PARPi monotherapy trials but only in the former resulted to be fatal in relation to one patient.^15^, ^16^, ^17^, ^18^, ^19^, ^20^, ^21^, ^22^, ^23^, ^24^, ^25^, ^26^ The mechanism of this effect is still unknown though it was assumed that it was not a drug‐class effect, but it could be related to COVID‐19 pandemic.^15^, ^16^, ^17^, ^18^, ^19^, ^20^, ^21^, ^22^, ^23^, ^24^, ^25^ However, in the combination therapy group, there was the necessity of temporary interruption of the therapy, dose reduction, and permanent discontinuation. Meanwhile, in the monotherapy group, the AE‐s peaked within the first 2 months of treatment and were generally manageable through dose modifications and supportive therapies without the need for treatment discontinuation, allowing patients to remain on treatment for as long as they were deriving clinical benefit.^15^, ^16^, ^17^

As described above, the heterogeneity of the selected studies either in first‐line or in pretreated settings represents a limitation for the present meta‐analysis: the difference in study design, enrolled patients population, especially for prior and/or subsequent treatments, as well as for molecular testing and gene‐selected preplanned analysis. Also, the mechanism of action among the different PARPi (olaparib and rucaparib inhibit PARP 1/2/3, whereas niraparib, talazoparib, and veliparib inhibit PARP 1/2 only) as well as their action/synergism with different ARTA (abiraterone, an androgen synthesis inhibitor while enzalutamide is an androgen receptor inhibitor) could justify the efficacy/safety differences among the trials.^27^

## 5 CONCLUSIONS

The present meta‐analysis highlights the benefit of PARPi‐based treatments, especially in terms of rPFS. The HRR+ population (and particularly BRCA2 and BRCA1 patients) had the most significant outcome benefit from PARP‐i. Safety analysis showed favourable toxicity profiles in combining PARP‐I and ARTA. PARPi‐based therapy represents a compelling treatment option for HRR+ mCRPC, mainly BRCA1/2‐mutated. However, further biomarker analysis is needed in order to identify other responsive patients across the different disease settings.

## CONFLICT OF INTEREST STATEMENT

Daniele Santini: Advisory Board role and speaker honoraria with AstraZeneca, MSD, BMS, Astellas, Janssenn, EISAI, Merck, Pfizer, Bayer, Novartis, AstraZeneca, Lilly. Michela Roberto: Advisory Board role and speaker honoraria with AstraZeneca, MSD, BMS, Astellas, Janssenn, Bayer, Novartis. All other authors declare no conflicts of interest.

## Supporting information


**Figure S1.** Risk of bias assessment
**Figure S2.** A. rPFS in studies comparing PARPi+ARTA vs ARTA in first line setting in patients with bone only metastases. B. rPFS in studies comparing PARPi+ARTA vs ARTA in first line setting in patients with visceral metastases
**Figure S3.** rPFS in studies comparing PARPi to TPC and PARPi+ARTA vs ARTA in second or subsequent lines in mCRPC.
**Figure S4.** Any grade treatment‐related adverse events in studies comparing PARPi combined with ARTA to ARTA alone
**Figure S5.** Grade > = 3 treatment‐related adverse events in studies comparing PARPi combined with ARTA and ADT to ARTA and ADT.
**Figure S6.** Any grade treatment‐related adverse events in studies comparing PARPi monotherapy to TPC.
**Figure S7.** Grade > = 3 treatment‐related adverse events in studies comparing PARPi monotherapy to TPC.
**Figure S8.** Main Adverse events in percentage reported in RCT on PARP inhibitors.
**Figure S9.** PRIMA checklist
